# Prevalence and Risk Factors Associated With Sexual Dysfunction in Malagasy Women With and Without Type 2 Diabetes Mellitus: A Cross‐Sectional Study in a University Hospital Center, Antananarivo Madagascar

**DOI:** 10.1002/hsr2.70267

**Published:** 2024-12-17

**Authors:** Sitraka Angelo Raharinavalona, Nomenjanahary Rakotoarimino, Miora Maëva Arielle Andrianiaina, Solofo Andrianarivelo Ralamboson, Radonirina Lazasoa Andrianasolo, Andrianirina Dave Patrick Rakotomalala

**Affiliations:** ^1^ Cardiovascular Diseases and Internal Medicine Departments Soavinandriana Hospital Center Antananarivo Madagascar; ^2^ Endocrinology Department Joseph Raseta Befelatanana University Hospital Center Antananarivo Madagascar

**Keywords:** female sexual dysfunction, prevalence, risk factors, type 2 diabetes mellitus

## Abstract

**Background and Aims:**

Female sexual dysfunction (FSD) remains a very little studied subject in Madagascar, despite the resulting alteration of quality of life. Our study aims to determine the prevalence and risk factors for sexual dysfunction in Malagasy women with and without type 2 diabetes (T2DM).

**Methods:**

This was a descriptive and analytical cross‐sectional study, carried out in the Endocrinology department of the Joseph Raseta Befelatanana University Hospital Center over a period of 18 months. FSD was assessed using the Female Sexual Function Index questionnaire in women with and without T2DM.

**Results:**

We retained 122 patients with T2DM and 127 without T2DM. The prevalence of FSD was 47.5% in diabetics and 44.1% in non‐diabetics. In patients with T2DM, factors associated with FSD were age [50–59 years] (OR = 2.03 [1.03–4.51]), nephropathy (OR = 2.18 [1.09–3.98]), ischemic stroke (*p* = 0.0483), ischemic heart disease (*p* = 0.005), carotid atherosclerosis (OR = 2.64 [1.09–7.85]), obesity (OR = 3.64 [1.22–12.3]), calcium channel blocker use (OR = 4.71 [1.35–21.0]), history of genitourinary infections (OR = 2.06 [1.07–4.28]). In patients without T2DM, they were age [50 – 59 years] (OR = 4.86 [2.16–11.3]), age of first sexual intercourse < 18 years (OR = 2.06 [1.04–4.57]), irregular menstrual cycle (OR = 2.02 [1.00–6.37]), number of gestations ≥ 4 (OR = 1.99 [1.00–5.41]), abortion (OR = 3.15 [1.21–29.1]) and number of children ≥ 4 (OR = 2.17 [1.01–4.81]).

**Conclusion:**

FSD was more common in diabetics than non‐diabetics. Its early management and associated risk factors are necessary to improve the quality of life of patients.

## 1 Introduction

According to the World Health Organization, sexual health is an integral part of overall health, well‐being and quality of life [1]. Female sexual dysfunction (FSD) can be defined as “any disturbance in sexual functioning involving one or more phases of the normal sexual response (excitement, plateau, orgasm, resolution) or associated pain [2, 3]. The overall prevalence of FSD is 40%–50% in premenopausal women, which most often involves desire and arousal [4, 5].

Particularly in diabetics, FSD is a multifactorial disorder involving subjective and objective aspects of sexual function, to which many factors (hormonal, psychological, interpersonal and social) contribute. Indeed, diabetes mellitus leads to various medical, psychological and sexual complications [6]. Thus, FSD is more common with a prevalence of up to 80%, mainly affecting patients with type 2 diabetes mellitus (T2DM) than type 1 diabetes mellitus [7, 8, 9].

FSD in patients with or without T2DM remain a subject that has been little studied or even taboo, especially in Sub‐Saharan Africa. They are also largely underdiagnosed and undertreated. While all sexual dysfunctions have negative effects on interpersonal and social relationships, as well as on the well‐being and quality of life of women [10, 11]. In Madagascar, a study has already been done on this subject in male diabetics. Nearly 8 out of 10 patients did not talk about their sexual dysfunction out of modesty and shame [12]. In women, this is one of the first studies carried out in our country.

The present study aims to determine the prevalence of female sexual dysfunction and its associated risk factors in Malagasy people with and without T2DM, to improve their care.

## 2 Materials and Methods

### 2.1 Study Design and Setting

A cross‐sectional analytical study was carried out over a period of 18 months, from September 1, 2020 to January 31, 2022, in the endocrinology department of the Joseph Raseta Befelatanana University Hospital Center, Antananarivo, the capital of Madagascar. It is the only public reference department for the management of metabolic and endocrine diseases in the country.

### 2.2 Study Population

Our study population is made up of two distinct and independent groups of patients: women with known and/or newly diagnosed T2DM and without T2DM. Patients aged less than 60 years, with or without FSD and followed in the study sitting, were included in the study. Those with a neurological disorder or mental illness making response conditions difficult, an incomplete record and refusing to participate, were excluded.

The diagnosis of known diabetes was made based on patient self‐report or taking antidiabetic drugs, that of newly diagnosed diabetes based on the diagnostic criteria of the American Diabetes Association [13]. T2DM was retained in view of the patient's age (> 35 years), overweight/obesity, family history of T2DM and the absence of obvious secondary causes (chronic pancreatitis, endocrinopathies, long‐term glucocorticoid use).

We assessed the presence of FSD according to the “Female sexual function Index (FSFI)” [14, 15]. The French version of this questionnaire was translated into Malagasy to make it easier for our patients to understand. It included 19 questions covering the areas of desire, arousal, lubrication, orgasm, satisfaction and pain. By adding the scores of the six domains, a total score less than 23 affirms FSD [16, 17]. Female sexual function was classified into four groups: normal female sexual function (total score ≥ 23), mild FSD (total score = 18–23), moderate FSD (total score = 11–17), and severe FSD (total score ≤ 10) [17].

The sampling of the population was exhaustive. The sample size was determined using a single population proportion formula: *n* = (*Z*_1‐*α*/2_) [2] P (1−*P*)/*d* ^2^); based on the assumptions of 95% confidence level (*Z*_1‐*α*/2_ = 1.96), 5% margin of error and a 25% proportion of sexual dysfunction. The prevalence of 25% was taken because there was no similar study done in Madagascar previously. The final sample size for the study was 288 women who consented to participate. However, 39 had not completed the survey forms correctly and/or their medical records were not complete. Thus, we retained 249 patients.

### 2.3 Clinical and Laboratory Data

The parameters studied were demographic data (age, educational status, housewife), characteristics of diabetes (duration, glycated hemoglobin, chronic degenerative complications), cardiovascular risk factors (hypertension, dyslipidemia, smoking, overweight, obesity), usual treatments (antihypertensives, statins, antiplatelet agents), gynecologic and obstetric history (age of first sexual intercourse, character of the menstrual cycle, menopause, number of births, abortions and children, Intrauterine device, pelvic surgery and urinary infections) and FSD.

The presence of hypertension was certified by blood pressure ≥ 140/90 mmHg (based on an average of ≥ 2 measurements obtained on ≥ 2 occasions) or taking antihypertensive medication. Patients with low density lipoprotein cholesterol (LDLc) levels outside the targets recommended by the European Society of Cardiology [18] or taking a lipid‐lowering agent were considered to have dyslipidemia. The body mass index was calculated as the weight in kilograms (kg) divided by the square of the height in meters (m^2^). Hb A1c was measured using the high‐performance liquid chromatography method.

### 2.4 Statistical Analysis

Data was collected from a pre‐established survey form from patient medical records. Then, they were used using Epi Info version 3.5.4 software (United States Centers for Disease Control and Prevention in Atlanta, Georgia). Qualitative and quantitative variables were respectively expressed as proportion and median with interquartile range [25% and 75%]. The chi‐square test with a significance threshold less than 0.05 and the odds ratio (OR) were used to determine the factors associated with FSD. This OR is affected by a 95% confidence interval [95%CI].

### 2.5 Ethical Considerations

This study had the approval of the Director of the University Hospital Center Joseph Raseta Befelatanana and the head of the endocrinology department, on August 28, 2020. Patients were previously informed about respect for confidentiality and anonymity which was preserved by a coding method. They had consented to participate in the study, by signing a pre‐established form.

## 3 Results

We retained 122 women with T2DM and 127 without T2DM. Their mean ages were 48.6 ± 6.45 years and 48.4 ± 6.43 years, respectively. Compared to those without T2DM, patients with T2DM had significantly more hypertension, overweight and obesity, received more than one renin‐angiotensin system (RAS) blockers and statin, more history of abortion and the use of an Intrauterine device as a method of contraception. Table 1 summarizes the general characteristics of the study population.

**Table 1:** General characteristics of the study population.

Variables	Total (*n* = 249)	With T2DM (*n* = 122)	Without T2DM (*n* = 127)	*p* value
**Mean age,** years (±SD)	48.5 (6.42)	48.6 (6.45)	48.4 (6.43)	0.2142
**Age groups,** years				
[30–39], *n* (%)	14 (5.6)	8 (6.6)	6 (4.7)	0.3623
[40–49], *n* (%)	124 (49.8)	58 (47.5)	66 (52.0)	0.2837
[50–59], *n* (%)	113 (45.4)	56 (45.9)	55 (43.3)	0.2934
**Educational status**				
None, *n* (%)	13 (5.2)	8 (6.6)	5 (3.9)	0.2602
Primary, *n* (%)	88 (35.3)	36 (29.5)	52 (41.0)	0.0394†
Secondary, *n* (%)	109 (43.8)	54 (44.2)	55 (43.3)	0.4903
Tertiary, *n* (%)	39 (15.7)	24 (19.7)	15 (11.8)	0.0625
**Housewife**, *n* (%)	79 (31.7)	42 (34.4)	37 (29.1)	0.2234
**Characteristics of diabetes**				
Mean duration, year (±SD)	—	3.6 (3.11)	—	—
Newly diagnosed diabetes, *n* (%)	—	48 (39.3)	—	—
Duration ≥ 5 years, *n* (%)	—	22 (18.0)	—	—
Mean glycated hemoglobin, (±SD)	—	11,7 (2.34)	—	—
Glycated hemoglobin ≥ 10%, *n* (%)	—	94 (77.0)	—	—
Associated chronic degenerative complications				
Nephropathy, *n* (%)	—	32 (26.2)	—	—
Neuropathy, *n* (%)	—	18 (14.8)	—	—
Retinopathy, *n* (%)	—	6 (4.9)	—	—
Ischemic stroke, *n* (%)	—	4 (3.3)	—	—
Ischemic heart disease, *n* (%)	—	6 (4.9)	—	—
Carotid atherosclerosis, *n* (%)	—	24 (19.7)	—	—
**Associated cardiovascular risk factors**				
Hypertension, *n* (%)	116 (46.6)	70 (57.4)	46 (36.2)	0.0004†
Dyslipidemia, *n* (%)	10 (4.0)	10 (8.2)	—	—
Smoking, *n* (%)	4 (1.6)	4 (3.3)	—	—
Overweight, *n* (%)	108 (43.4)	60 (49.2)	48 (37.8)	0.0459†
Obesity, *n* (%)	28 (11.2)	22 (18.0)	6 (4.7)	0.0007†
**Other previous treatments**				
Calcium channel blockers, *n* (%)	39 (15.7)	18 (14.8)	21 (16.5)	0.4163
RAS blockers, *n* (%)	66 (26.5)	42 (34.4)	24 (18.9)	0.0145†
Diuretics, *n* (%)	12 (4.8)	2 (1.6)	10 (7.9)	0.0202†
Statins, *n* (%)	18 (7.3)	18 (15.0)	0 (0)	< 0.0001†
**Gynecological–obstetric history**				
Age of first sexual intercourse < 18 years, *n* (%)	99 (39.8)	50 (41.0)	49 (38.6)	0.3984
Irregular menstrual cycle, *n* (%)	56 (22.5)	28 (23.0)	28 (22.0)	0.4922
Number of gestations ≥ 4, *n* (%)	138 (55.4)	66 (54.1)	72 (56.7)	0.3881
Abortion, *n* (%)	37 (14.9)	24 (19.7)	13 (10.2)	0.0274†
Number of children ≥ 4, *n* (%)	130 (52.2)	62 (50.8)	68 (53.5)	0.3809
Intrauterine device, *n* (%)	14 (5.6)	12 (9.8)	2 (1.6)	0.0023†
Pelvic surgery, *n* (%)	35 (14.1)	16 (13.1)	19 (15.0)	0.4069
Genitourinary infections, *n* (%)	103 (41.4)	54 (44.3)	49 (38.6)	0.2174

Abbreviations: RAS, renin–angiotensin system; SD, Standard deviation; T2DM, type 2 diabetes mellitus.

^†^ *p* < 0.05.

The prevalence of FSD was 47.5% in patients with T2DM and 44.1% in those without T2DM (Table 2). The proportions of sexual dysfunction according to their severity were not statistically different.

**Table 2:** Sexual function of Malagasy women with and without type 2 diabetes mellitus.

Variables	Total (*n* = 249)	With T2DM (*n* = 122)	Without T2DM (*n* = 127)	*p* value
Normal sexual function, *n* (%)				
Sexual dysfunction, *n* (%)	114 (45.7)	58 (47.5)	56 (44.1)	0.3378
Mild sexual dysfunction, *n* (%)	27 (10.8)	10 (8.2)	17 (13.4)	0.1328
Moderate sexual dysfunction, *n* (%)	7 (2.8)	6 (4.9)	1 (0.8)	0.0548
Severe sexual dysfunction, *n* (%)	80 (32.1)	42 (34.4)	38 (29.9)	0.2659

Abbreviation: T2DM, type 2 diabetes mellitus.

In patients with T2DM, FSD was significantly associated with age [50–59 years] (OR = 2.03 [1.03–4.51], *p* = 0.0378), nephropathy (*p* = 2.18 [1.09–3.98], *p* = 0.0252), ischemic stroke (*p* = 0.0483), coronary artery disease (*p* = 0.005), carotid atherosclerosis (OR = 2.64 [1.09–7.85], *p* = 0.0307), obesity (OR = 3.64 [1.22–12.3], *p* = 0.0051), taking a calcium channel blocker (OR = 4.71 [1.35–21.0], *p* = 0.0031), using an Intrauterine device (OR = 0.19 [0.02–0.97], *p* = 0.0228), history of genitourinary infections (OR = 2.06 [1.07–4.28], *p* = 0.0292). Table 3 summarized the factors associated with female sexual dysfunction in patients with T2DM.

**Table 3:** Factors associated with female sexual dysfunction in patients with type 2 diabetes mellitus.

Variables	With FSD (*n* = 58)	Without FSD (*n* = 64)	OR [95% CI]	*p* value
**Mean age**, years (±SD)	49.9 (5.52)	46.7 (4.94)	—	0.0030†
**Age groups**, years				
[30–39], *n* (%)	2 (3.4)	6 (9.4)	0.34 [0.03–2.05]	0.1707
[40–49], *n* (%)	24 (41.4)	34 (53.1)	0.62 [0.29–1.35]	0.1322
[50–59], *n* (%)	32 (55.2)	24 (37.5)	2.03 [1.03–4.51]	0.0378†
**Educational status**				
None, *n* (%)	4 (6.9)	4 (6.3)	1.11 [0.19–6.26]	0.5849
Primary, *n* (%)	18 (31.0)	18 (28.1)	1.14 [0.49–2.69]	0.4387
Secondary, *n* (%)	26 (44.8)	28 (43.8)	1.04 [0.48–2.27]	0.5248
Tertiary, *n* (%)	10 (17.2)	14 (21.9)	0.74 [0.26–2.01]	0.3401
**Housewife**, *n* (%)	16 (27.6)	26 (40.6)	0.56 [0.24–1.27]	0.0926
**Characteristics of diabetes**				
Mean duration, year (±SD)	3.9 (3.44)	4.2 (3.13)	—	0,0528
Duration ≥ 5 years, *n* (%)	8 (13.8)	14 (21.9)	0.57 [0.19–1.62]	0.1781
Mean glycated hemoglobin, (±SD)	11.6 (3.01)	11.8 (2.87)	—	0.0001†
Glycated hemoglobin ≥ 10%, *n* (%)	44 (75.9)	50 (78.1)	0.88 [0.34–2.23]	0.4669
Associated chronic degenerative complications				—
Nephropathy, *n* (%)	22 (37.9)	10 (15.6)	2.18 [1.09–3.98]	0.0252†
Neuropathy, *n* (%)	10 (17.2)	8 (12.5)	1.45 [0.47–4.61]	0.3146
Retinopathy, *n* (%)	0 (0)	6 (5.0)	ND	0.9036
Ischemic stroke, *n* (%)	4 (6.9)	0 (0)	ND	0.0483†
Ischemic heart disease, *n* (%)	6 (10.3)	0 (0)	ND	0.005†
Carotid atherosclerosis, *n* (%)	16 (27.6)	8 (12.5)	2.64 [1.09–7.85]	0.0307†
**Associated cardiovascular risk factors**				
Hypertension, *n* (%)	34 (58.6)	36 (56.3)	1.13 [0.41–1.97]	0.4678
Dyslipidemia, *n* (%)	6 (10,3)	4 (6.3)	1.72 [0.38–8.77]	0.3108
Smoking, *n* (%)	2 (3.4)	2 (3.1)	1.11 [0.08–15.7]	0.6525
Overweight, *n* (%)	22 (37.9)	38 (59.4)	0.42 [0.19–0.92]	0.0142†
Obesity, *n* (%)	16 (27.6)	6 (9.4)	3.64 [1.22–12.3]	0.0051†
**Other previous treatments**				
Calcium channel blockers, *n* (%)	14 (24.1)	4 (6.3)	4.71 [1.35–21.0]	0.0031†
RAS blockers, *n* (%)	20 (34.5)	22 (34.4)	1.00 [0.44–2.26]	0.5703
Statins, *n* (%)	4 (7.1)	14 (21.9)	0.27 [0.06–0.96]	0.021†
**Gynecological–obstetric history**				
Age of first sexual intercourse < 18 years, *n* (%)	22 (37.9)	28 (43.8)	0.78 [0.35–1.72]	0.3201
Irregular menstrual cycle, *n* (%)	14 (24.1)	14 (21.9)	1.13 [0.44–2.88]	0.4669
Number of gestations ≥ 4, *n* (%)	32 (55.2)	34 (53.1)	1.09 [0.50–2.35]	0.4823
Abortion, *n* (%)	10 (17.2)	14 (21.9)	0.75 [0.26–2.01]	0.3401
Number of children ≥ 4, *n* (%)	28 (48.3)	34 (53.1)	0.82 [0.38–1.78]	0.3618
Intrauterine device, *n* (%)	2 (3.4)	10 (15.6)	0.19 [0.02–0.97]	0.0228†
Pelvic surgery, *n* (%)	6 (10.3)	10 (15.6)	0.62 [0.17–2.06]	0.2775
Genitourinary infections, *n* (%)	34 (53.1)	20 (34.5)	2.06 [1.07–4.28]	0.0292†

Abbreviations: CI, Confidence Interval; FSD, female sexual dysfunction; OR, odds ratio; RAS, renin–angiotensin system; SD, standard deviation.

^†^ *p* < 0.05.

In patients without T2DM, the factors significantly associated with FSD were age [50–59 years] (OR = 4.86 [2.16–11.3], *p* < 0.0001), overweight (OR = 0.49 [0.21–0.98], *p* = 0.0422), age of first sexual intercourse ≥ 18 years (OR = 2.06 [1.04–4.57]), *p* = 0.0362), irregular menstrual cycle (OR = 2.02 [1.00–6.37], *p* = 0.0472), the number of gestations ≥ 4 (OR = 1.99 [1.00–5.41], *p* = 0.0429), abortion (OR = 3.15 [1.21–29.1], *p* = 0.0248) and the number of children ≥ 4 (OR = 2.17 [1.01–4.81], *p* = 0.0237). Table 4 summarizes the factors associated with female sexual dysfunction in patients without T2DM.

**Table 4:** Factors associated with female sexual dysfunction in patients without type 2 diabetes mellitus.

Variables	With FSD (*n* = 56)	Without FSD (*n* = 71)	OR [95% CI]	*p* value
**Mean age**, years (±SD)	48.5 (6.42)	46.4 (5.23)	—	0.0275†
**Age groups**, years				
[30–39], *n* (%)	0 (0)	6 (9.4)	ND	0.0277†
[40–49], *n* (%)	20 (35.7)	46 (64.8)	0.31 [0.13–0.66]	0.0006†
[50–59], *n* (%)	36 (64.3)	19 (26.8)	4.86 [2.16–11.3]	< 0.0001†
**Educational status**				
None, *n* (%)	2 (3.6)	3 (4.2)	0.84 [0.06–7.61]	0.6114
Primary, *n* (%)	24 (42.9)	28 (39.4)	1.15 [0.53–2.49]	0.4174
Secondary, *n* (%)	25 (44.6)	30 (42.3)	1.10 [0.51–2.37]	0.4640
Tertiary, *n* (%)	5 (14.1)	10 (14.1)	0.60 [0.15–2.07]	0.2711
**Housewife**, *n* (%)	16 (28.6)	21 (29.6)	0.95 [0.41–2.21]	0.5302
**Associated cardiovascular risk factors**				
Hypertension, *n* (%)	19 (33.9)	27 (38.0)	0.93 [0.54–2.66]	0.3862
Overweight, *n* (%)	16 (28.6)	32 (45.1)	0.49 [0.21–0.98]	0.0422†
Obesity, *n* (%)	2 (3.6)	4 (5.6)	0.62 [0.05–4.53]	0.4582
**Other previous treatments**				
Calcium channel blockers, *n* (%)	7 (12.5)	14 (19.7)	0.58 [0.18–1.69]	0.1993
RAS blockers, *n* (%)	12 (21.4)	13 (18.3)	1.21 [0.46–3.21]	0.4134
**Gynecological – obstetric history**				
Age of first sexual intercourse < 18 years, *n* (%)	27 (48.2)	22 (31.0)	2.06 [1.04–4.57]	0.0362†
Irregular menstrual cycle, *n* (%)	20 (28.2)	8 (14.3)	2.02 [1.00–6.37]	0.0472†
Number of gestations ≥ 4, *n* (%)	37 (66.1)	35 (49.3)	1.99 [1.00–5.41]	0.0429†
Abortion, *n* (%)	11 (15.5)	2 (3.6)	3.15 [1.21–29.1]	0.0248†
Number of children ≥ 4, *n* (%)	36 (64.3)	32 (45.1)	2.17 [1.01–4.81]	0.0237†
Intrauterine device, *n* (%)	2 (3.6)	0 (0)	ND	0.1924
Pelvic surgery, *n* (%)	8 (14.3)	11 (15.5)	0.91 [0.29–2.71]	0.5271
Genitourinary infections, *n* (%)	32 (45.1)	17 (30.4)	1.26 [0.87–3.17]	0.0653

Abbreviations: CI, confidence Interval; FSD, female sexual dysfunction; OR, odds ratio; RAS, renin–angiotensin system; SD, standard deviation; T2DM, type 2 diabetes mellitus.

^†^ *p* < 0.05

## 4 Discussion

The prevalence of FSD varied between studies and countries. Among diabetics, it was 47.5% in the present study, fluctuated between 17% and 53.6% in Western countries [19, 20, 21], up to 79.2% in Eastern and Middle Eastern countries [22, 23, 24] and 88% in African countries [25, 26, 27]. It was the same in non‐diabetics, with a prevalence of 44.1% in the present study, from 28% to 63% in other studies [28, 29, 30]. This disparity could be explained not only by the difference in study population and culture of each country, but also the FSFI versions, and especially the difference in FSD diagnostic threshold value. Regardless, FSD is common in both diabetics and non‐diabetics.

In the present study, although the statistical test was not significant, FSD was more severe in patients with than without T2DM. What also observed in the literature [22]. Indeed, diabetes is associated with mental health problems, altering libido. Complications (neuropathy, vascular diseases, and genitourinary infections) contribute to the occurrence of FSD [31]. In addition, diabetics generally have other cardiovascular risk factors which constitute predictive factors for FSD [32].

Altering the different phases of the female sexual response, advanced age is a risk factor for FSD in both diabetics and non‐diabetics [17, 33]. This was also observed in the present study. Furthermore, the education status of women influences their quality of sexual life, since science and knowledge play a role on the intellectual state of individuals [34]. In an Ethiopian study, the risk of sexual dysfunction was 3.2 times higher for patients who had not attended school compared to those who had attended secondary school or higher [25]. The duration and control of diabetes did not influence the occurrence of FSD in the present study as in other studies [19, 35, 36].

In our patients with T2DM, other factors associated with FSD were nephropathy, ischemic stroke, coronary artery disease, carotid atherosclerosis, obesity, and history of genitourinary infections. Likewise, in the study by Li et al. in China, cerebrovascular and cardiovascular complications of diabetes were risk factors for FSD [22]. Also, the association between microvascular complications and FSD is significant, especially in patients with T2DM [37, 38]. According to a meta‐analysis by Dilixiati et al. patients with cardiovascular disease are at higher risk of developing female sexual dysfunction [39]. Abu Ali et al. found that a higher body mass index was significantly associated with less sexual function [40]. Dyslipidemia is an independent risk factor for FSD according to the literature [41]. Indeed, high chemerin/low adiponectin ratio plays a vital role in causing dyslipidemia and metabolic syndrome in patients, which impairs gonadal function, leading to FSD and others diabetes complications [42, 43]. According to Scavello et al. increased vascular resistance of the clitoral and uterine arteries on Doppler ultrasound is associated with cardiometabolic risk factors in women with sexual symptoms [44]. Furthermore, hyperglycemia increases the risk of genitourinary infections which, in turn, can lead to vaginal discomfort and dyspareunia hence FSD [38]. Regarding drugs intake, beta‐blockers are the only antihypertensive agents with relatively strong evidence of damage to female sexual function [45]. The association between the calcium channel blocker and FSD in this study still deserves to be further explored in future studies.

For our nondiabetic patients, age at first sexual intercourse < 18 years, irregular menstrual cycle, number of births ≥ 4, abortion and number of children ≥ 4 constituted risk factors for FSD. Similarly, in the Iranian study, the proportion of women who had first sexual intercourse before the age of 18 was significantly higher among those with than without FSD, and inversely for those from the age of 20. The number of children > 2 was a predictive factor for desire and orgasm disorders [17]. An Ethiopian study also found that irregular menstrual cycle was a risk factor for FSD after a bivariate analysis [29]. In a Nigerian series, the associations between the prevalence of FSD and age at coitarche, number of living children, and parity were significant [46]. The number of deliveries could lead to pelvic floor dysfunction which will be the origin of FSD [47, 48]. A systematic review and meta‐analysis by Jorge et al. showed that pelvic floor muscle training improved female Female Sexual Function Index total score [49]. A recent study highlights the importance of strengthening medical education of FSD from undergraduate of the curriculum, to improve students' comfort with the subject and to minimize the occurrence of FSD [50]. Thus, sex education for young women would seem essential, especially in countries like ours where sexuality remains a taboo subject.

This study will already serve to compact the databases in Madagascar on the sexuality of women with and without T2DM, and above all to improve their care. It also encourages the initiation of national study. Despite everything, it has limitations, notably the veracity of the responses in relation to the self‐report of problems with sexual activities. As risk factors for FSD, intrapsychic and relational factors were not assessed. Due to the monocentricity of the study, the results we obtained cannot be extrapolated to the entire general population.

## 5 Conclusion

The present study confirmed the high prevalence of FSD in patients with and without T2DM. The common risk factor of two groups was advanced age. Those of diabetics were dominated by vascular and infectious complications, and obesity, while those of non‐diabetics by gynecological – obstetric history. Early detection of FSD and these risk factors will therefore be essential, to plan adequate psychological and medical care, and to reduce the occurrence of this disorder.

## Author Contributions

**Sitraka Angelo Raharinavalona:** conceptualization, investigation, writing–original draft, methodology, visualization, validation, writing–review and editing, software, formal analysis, project administration, data curation, resources. **Nomenjanahary Rakotoarimino:** Investigation, conceptualization, methodology, data curation. **Miora Maëva Arielle Andrianiaina:** Investigation. **Solofo Andrianarivelo Ralamboson:** validation, writing–review and editing. **Radonirina Lazasoa Andrianasolo:** validation, writing–review and editing, supervision. **Andrianirina Dave Patrick Rakotomalala:** validation, writing–review and editing, supervision.

## Conflicts of Interest

The authors declare no conflicts of interest.

## Transparency Statement

The lead author Sitraka Angelo Raharinavalona affirms that this manuscript is an honest, accurate, and transparent account of the study being reported; that no important aspects of the study have been omitted; and that any discrepancies from the study as planned (and, if relevant, registered) have been explained.

## Data Availability

The datasets used and/or analyzed during the current study are available from the corresponding author upon reasonable request.
